# SKESA: strategic k-mer extension for scrupulous assemblies

**DOI:** 10.1186/s13059-018-1540-z

**Published:** 2018-10-04

**Authors:** Alexandre Souvorov, Richa Agarwala, David J. Lipman

**Affiliations:** 1 NCBI/NLM/NIH/DHHS, 8600 Rockville Pike, Bethesda, 20894 MD USA; 2 Impossible Foods, impossiblefoods.com, Redwood City, 94063 CA USA

**Keywords:** Illumina reads, De-novo assembly, DeBruijn graphs, Sequence quality, Contamination

## Abstract

SKESA is a DeBruijn graph-based de-novo assembler designed for assembling reads of microbial genomes sequenced using Illumina. Comparison with SPAdes and MegaHit shows that SKESA produces assemblies that have high sequence quality and contiguity, handles low-level contamination in reads, is fast, and produces an identical assembly for the same input when assembled multiple times with the same or different compute resources. SKESA has been used for assembling over 272,000 read sets in the Sequence Read Archive at NCBI and for real-time pathogen detection. Source code for SKESA is freely available at https://github.com/ncbi/SKESA/releases.

**Electronic supplementary material:**

The online version of this article (10.1186/s13059-018-1540-z) contains supplementary material, which is available to authorized users.

## Background

Sequence alignment, assembly, variation detection, or some combination thereof are usually the major modules of any bioinformatics pipeline analyzing next-generation sequence (NGS) read data [1–6]. An important application for microbial genome sequencing is to detect pathogenic outbreaks in the food supply chain [7–9] and in hospitals [10–13]. Advantages and bioinformatics challenges in using NGS for surveillance and outbreak investigations of foodborne pathogens were reviewed using *Listeria Monocytogenes* as an example [14] and by citing retrospective and real-time outbreaks [15]. Both reviews identified de-novo assembly of NGS as a significant challenge in using the information.

A collaboration between US states, federal agencies, and international partners to deposit foodborne bacterial pathogen sequence data at the National Center for Biotechnology Information (NCBI), referred to as the Pathogen Detection Project (PDP), has accelerated NGS-based investigations of outbreaks. The number of read sets submitted for the four major species of foodborne pathogens, namely, Salmonella, Listeria, Escherichia and Shigella, and Campylobacter, have scaled rapidly from 44011 to 85823 to 145178 read sets in the results published by PDP at the beginning of 2016, 2017, and 2018, respectively. More outbreaks are now identified when clusters are still small and fewer people are affected [11, 16]. The dominant sequencing technology in PDP is Illumina that has very low insertion deletion error rate but suffers from some systematic biases and low-level carryover contamination from earlier runs [17–20].

Several de-novo assemblers for sequence reads have been published [21–28]. Some are specialized for ploidity [29], metagenomes [30–35], single cell [36], sequencing technologies [37], or combine several assemblies into one [38]. No assembler guarantees an “error-free” assembly even for haploid genomes. In addition to microbial genomes, haploid assemblies are of interest for special human genome cases, such as from a hydatidiform mole [39]. Some applications for microbial genomes, such as PDP, rely on vertical inheritance of genomic data from mother to daughter cell and resolve patterns of read set clonality based on very few variations, typically less than 10 variations in a 4 Mb genome [15]. Such applications require assemblies with very high sequence quality so that true variations can be detected with confidence.

Assessment of assemblies using read sets generated by sequencing machines requires a publicly available benchmarking set that contains both the reads and a near complete high-quality draft assembly for the same sample. FDA-ARGOS is a database developed by the Food and Drug Administration (US FDA) [19] that consists of regulatory grade sequences for microbes and satisfies the benchmark requirements for assembly quality assessment of microbial genomes.

Here, we focus on the problem of quickly computing a high-quality de-novo sequence assembly of reads from microbial genomes generated using Illumina sequencing technology and present our de-novo assembler called SKESA [*skee–sa*] (strategic k-mer extension for scrupulous assemblies). Heuristics used by SKESA are designed to reduce the effect of low-level contamination and strand specific errors in Illumina sequencing on the quality of the assembly. For other sequencing technologies with high error rates, conservative heuristics used by SKESA will create less contiguous assemblies than those generated by some other assemblers. SKESA can assemble genomes larger than microbial genomes but has not been profiled or compared to other assemblers for such genomes. For example, SKESA assembles SRR7262862 (49.8 million reads with total length 9.2 Gb) for *Monilinia fructigena* and SRR6748693 (73.2 million reads with total length 18.3 Gb) for *Monilinia laxa* where assemblies are ∼ 40 Mb long in under 3 h with 10 cores and under 30 min with 100 cores.

Many de-novo assemblers, including SPAdes [24] and MegaHit [35], use DeBruijn graphs and multiple k-mer lengths during assembly. ALLPATHS_LG [40] used a specific short insert library construction protocol where 100 bp mates overlapped by 40 bases. Using the overlap, they produced 160 bp merged reads but only used 96 as the largest k-mer size for their assembly. The distinguishing feature of SKESA is that it generates k-mers that are longer than mates and up to insert size from mini-assemblies of a subset of reads. This feature of using longer than mate length k-mers allows SKESA to assemble regions accurately that have repeats shorter than insert size but longer than the mate length. To our knowledge, all current assemblers, in contrast, only use k-mers up to the size of mates.

In this manuscript, we compare SKESA to SPAdes and MegaHit using five types of microbial test sets [41]: (i) *run time set (RTS)* that has 56 read sets identified by the PDP team, (ii) *benchmark set* that has 403 read sets from FDA-ARGOS, (iii) *random set* that has 5000 randomly chosen read sets from Sequence Read Archive (SRA), (iv) *contamination set* that is a simulated set with six read sets at different levels of contamination, and (v) *substrings set* that is also a simulated set with 131 read sets at different lengths of substrings of a reference genome. These sets provide a total of 6044 runs for each assembly method as each read set in the *RTS* set was run three times each and on three different compute resource settings. A full description of the test sets is given in the “Methods” section. We chose MegaHit and SPAdes for comparison as MegaHit is a very fast assembler and SPAdes is a versatile and widely used assembler that provides options for various technologies and sample types. Results that also include comparison to IDBA [36] (version 1.1.1) designed to handle uneven coverage of genome by reads and to ABySS [28] (version 2.0.2) designed to assemble both large and small genomes are available in supplementary material (Additional file 1) but are not discussed in the main manuscript as the implication of these results is same as the one we get using MegaHit and SPAdes.

We show that for assemblies of microbial genomes, SKESA and MegaHit are comparable in speed and significantly faster than SPAdes. SKESA can also access reads directly from SRA and doing so is faster than reading the input from files. Assembly quality measured by the number of mismatches per 100 Kb as computed by QUAST [42], assembly contiguity as measured by the N50 statistic, and deviation from the length of the reference assembly show that quality of SKESA assemblies is better than that of SPAdes and MegaHit. On the same input, SKESA produces identical results regardless of the number of threads, memory, or the number of times runs are done. This is a critical requirement for production systems that handle large volumes of data and require regression tests. In our tests, both SPAdes and MegaHit produce different assemblies across iterations, even for the same setting of number of threads and memory. Therefore, SKESA meets all the requirements for producing microbial assemblies needed for applications such as PDP where assemblers are required to produce assemblies that have high base level sequence quality and contiguity sufficient for downstream analysis, handle low-level contamination in reads, and be fast and robust in production environments. SKESA is currently used in production at NCBI for assembling microbial genomes for SRA and has been incorporated into the workflow of PDP. Software for SKESA is freely available [43, 44] (see “Availability and requirements”) and will also be made available in the cloud.

## Results and discussion

### Production usage

As of March 2018, SKESA had been used by NCBI to assemble over 272,000 read sets available in SRA including assemblies for Salmonella (131,581 assemblies), Listeria (19,718 assemblies), Escherichia (65,307 assemblies), Shigella (10,942 assemblies), Campylobacter (32,416 assemblies), and Clostridioides (12,042 assemblies). These species are of importance for detecting pathogens in the food supply chain and in hospitals. Assemblies are publicly available in a downloadable object for each read set from the SRA website.

### Computation time

For read sets in the *RTS* set, Table 1 shows median wall-clock time and distribution of wall-clock time by method and compute resource settings where input is read from files. For each read set *R*, each method *M*, and each resource setting *S*, assembly was performed three times and the minimum of the three wall-clock times was taken as the time reported for that combination of *R*,*M*, and *S*. Results for the median wall-clock time show that all methods scale well with increase in compute resources. The distribution of wall-clock time shows that MegaHit is fastest with SKESA being a close second, but SPAdes is substantially slower. SKESA is faster when reads are accessed directly from SRA (data not shown).

**Table 1:** Run time comparison using 56 inputs in the run time set

Run time	4 cores, 16 Gb			8 cores, 32 Gb			12 cores, 32 Gb		
(seconds)	SKESA	SPAdes	MegaHit	SKESA	SPAdes	MegaHit	SKESA	SPAdes	MegaHit
<=300	6	1	6	16	2	24	32	3	37
301−400	3	0	2	16	1	12	11	3	11
401−500	5	2	8	7	3	7	2	1	5
501−600	6	1	10	6	1	8	3	3	0
601−700	10	1	6	0	3	2	3	3	0
> 700	26	51	24	11	46	3	5	43	3
Median	688	2303	616	359	1319	328	275	1086	240

Best of three wall-clock times is used for each input, method, and resource combination

### Software robustness

SKESA and MegaHit were successful in assembling all read sets in all test sets under all settings of compute resources used. SPAdes did not produce an assembly for 23 out of 6044 runs. These were (i) three runs for read set SRR1515967 in the *RTS* set done using 4 cores and 16 Gb memory, (ii) 18 read sets from the benchmark set even with 100 cores and 250 Gb memory, and (iii) read sets at k-mer length 34 and 56 in the substrings set. In addition, assembly for 10 read sets from the random set using SPAdes required more than 16 Gb whereas SKESA and MegaHit were successful in assembling them with the 16-Gb memory limit.

SKESA produces the same assembly for a read set regardless of the number of times assembly is performed, number of cores, or memory available. The same does not hold true for MegaHit and SPAdes. As an example, with MegaHit, all nine runs for read set SRR2820668 in the run time set produced the same N50 of 101,087 bp but different number of contigs (172 to 178) and nine different sizes of assembly (6,872,670 bp to 6,874,132 bp). An example where SPAdes produced different number of contigs and assembly sizes for the same read set and same settings of resources is SRR1515967. In three runs for SRR1515967 with 12 cores and 32 Gb memory, SPAdes produced 1937 contigs with assembly size 5,553,327 and N50 of 135,184 bp, 1952 contigs with assembly size 5,555,233 and N50 of 154,465 bp, and 1927 contigs with assembly size 5,552,535 and N50 of 115,121 bp. We note that for the 56 read sets in the *RTS* set, MegaHit did not produce an identical assembly in all nine runs for any of the read sets while SPAdes did so for 12 read sets.

### Sequence quality

For the benchmark set and each assembly method, the number of misassemblies (Table 2), number of mismatches per 100 Kb (Table 3), deviation statistics (Table 4), and contiguity statistics (Table 5) show that SKESA has a lower number of misassemblies, better base level sequence correctness, lower deviation from the length of reference, and contiguity comparable to that of SPAdes and MegaHit.

**Table 2:** Number of misassemblies in 381 inputs in the benchmark set

Count	SKESA	SPAdes	MegaHit
0	214	172	128
1	83	98	91
2	40	43	66
3	13	30	30
4	9	12	18
5	7	7	15
6	2	3	10
7	2	0	5
8	1	1	3
9	0	0	2
10+	10	15	13
Median	0	1	1

**Table 3:** Mismatches per 100 Kb as reported by QUAST for benchmark and contamination sets

Benchmark set			
Measure	SKESA	SPAdes	MegaHit
Median	0.08	2.76	1.89
Maximum	7.78	41.60	31.94
Average	0.40	3.21	2.79
Assembly counts in benchmark set			
Mismatches range	SKESA	SPAdes	MegaHit
0	105	1	1
0.01−1	247	40	80
1.01−2	9	76	121
2.01−3	9	89	58
3.01−4	1	71	45
> 4	10	104	76
Mismatches reported in contamination set			
Set	SKESA	SPAdes	MegaHit
No contamination	0	1.44	3.83
3x contamination	0	1.42	3.21
6x contamination	0	1.44	3.02
9x contamination	0.02	1.61	4.38
12x contamination	0.02	1.52	4.96
15x contamination	0.04	1.50	5.83

**Table 4:** Deviation of assembly length produced by the assemblers from the assembly length of the reference as computed using aligned length reported by QUAST and assembly lengths for benchmark and contamination sets

Benchmark set			
Measure	SKESA	SPAdes	MegaHit
Median	2.72	10.91	5.59
Maximum	135.75	775.14	407.78
Average	4.61	57.98	24.23
Deviation in contamination set			
Contamination	SKESA	SPAdes	MegaHit
None	1.33	1.68	1.35
3x	1.36	1.68	1.33
6x	1.33	1.68	1.30
9x	1.36	1.67	1.47
12x	1.41	1.68	2.05
15x	1.44	1.68	2.96

**Table 5:** Contiguity for benchmark, random, and contamination sets

Benchmark set			
N50 measure	SKESA	SPAdes	MegaHit
<=10 Kb	14	69	19
10001−50 Kb	40	41	46
50001−100 Kb	41	56	67
100001−250 Kb	191	169	197
250001−500 Kb	77	43	48
> 500 Kb	18	3	4
Median	170,647	117,340	124,833
Minimum	1832	364	687
Maximum	1,197,860	622,367	617,087
Average	195,141	131,823	146,706
N50 statistic in contamination set			
Contamination	SKESA	SPAdes	MegaHit
None	282,763	260,531	202,384
3x	282,763	260,531	202,384
6x	282,763	260,532	202,384
9x	225,630	260,531	151,916
12x	77,455	260,531	107,175
15x	42,440	260,531	65,124
Random set			
N50 measure	SKESA	SPAdes	MegaHit
<=10 Kb	6	10	6
10001−50 Kb	349	206	285
50001−100 Kb	788	409	1516
100001−250 Kb	2307	2369	2889
250001−500 Kb	1324	1616	266
> 500 Kb	226	390	38
Median	170,877	208,907	117,074
Minimum	2414	209	4182
Maximum	1,545,488	1,530,182	1,499,532
Average	213,847	255,079	136,339

For the contamination set, SKESA has no misassemblies, SPAdes has one misassembly for all inputs, and MegaHit has one misassembly for all inputs except the one at 15x where it has two misassemblies. The number of mismatches per 100 Kb (Table 3) and contiguity statistics (Table 5) show that SKESA suffers the most in contiguity when contamination level increases to 9x or above but maintains good base level accuracy, SPAdes maintains contiguity at an increased rate of base level inaccuracies, and MegaHit loses some contiguity as well as accuracy. Table 4 shows that assembly lengths of SKESA assemblies have the least total deviation across all sets, that assembly lengths of SPAdes assemblies do not depend on contamination, and that assembly lengths of MegaHit assemblies become most deviant at higher levels of contamination.

Contiguity statistics for the random set (Table 5) show that all methods produce good contiguity for most sets with SPAdes giving the best overall contiguity.

**Fig. 1:**
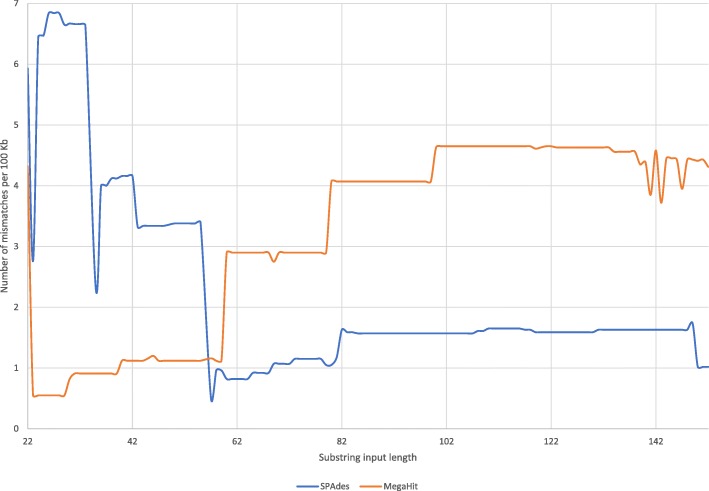
Substrings mismatches: mismatches per 100 Kb seen in assemblies of SPAdes and MegaHit for inputs in substrings set. SKESA has no mismatches at any length in this set

**Fig. 2:**
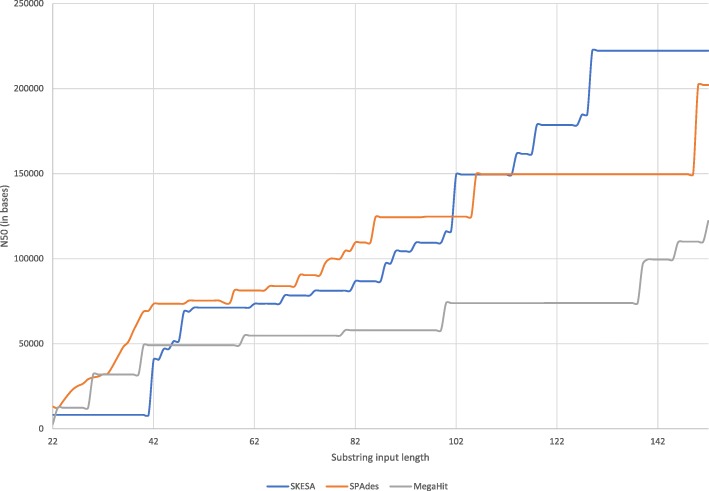
Substrings contiguity: N50 for assemblies generated by SKESA, SPAdes, and MegaHit for inputs in substrings set

**Fig. 3:**
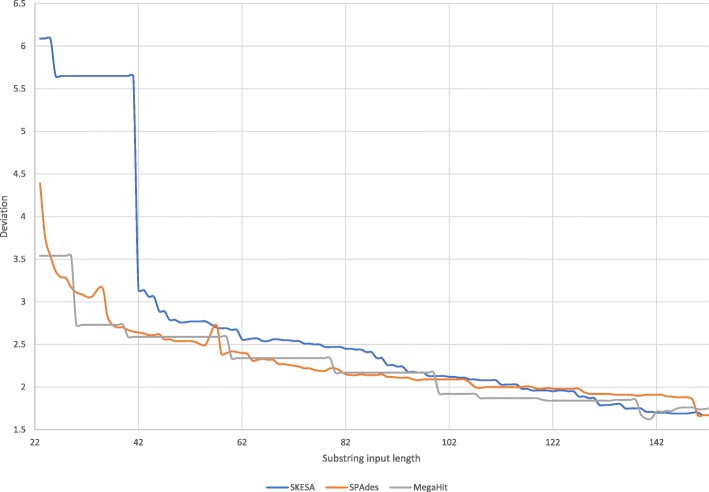
Substrings deviation: deviation for assemblies generated by SKESA, SPAdes, and MegaHit for inputs in substrings set. We do not show values for input length 22 where MegaHit has value of almost 100 and input length 34 and 56 for which SPAdes did not produce an assembly

For the substrings set that has single reads, SKESA has no misassemblies, SPAdes has nine misassemblies for the read set generated with length 22, and MegaHit has at least one misassembly for all read sets generated with length 60 or above. With MegaHit, nine inputs have two misassemblies and assembly of the read set with longest reads has three misassemblies. SKESA also has no mismatches while both SPAdes and MegaHit have mismatches as shown in Fig. 1. SKESA starts out with smallest contiguity at short read lengths but has highest contiguity at longer read lengths as shown in Fig. 2. SKESA also starts out with most deviation from the reference assembly length but becomes least deviant at inputs with longer reads as shown in Fig. 3.

### Read trimming

No read sets in benchmark, contamination, or substrings set were trimmed. Only 5 out of 56 read sets in the *RTS* set and 219 out of 5000 read sets in the random set were trimmed. We compared the most frequent k-mer marked as suspect in each of the 224 runs with known Illumina adaptors. All five in the *RTS* set and 199 in the random set had AGATGTGTATAAGAGACAG as the most frequent k-mer that is a known Illumina adaptor (Nextera and others). The remaining 20 included 12 that consisted of homopolymer C, 5 that consisted of homopolymer A, one that is a TrueSeq adaptor (AGATCGGAAGAGCGTCGTG), and one each that were ATCAAAGGAAATGATAGCA (in SRR5221560) and CTTTTTTGGTGCTTTAGCA (in SRR5414541). It appears to us that the k-mers found as suspected in SRR5221560 could be from a cloning vector and confirm that a plasmid is not produced in SKESA assembly of SRR5414541 because of read trimming. In all read sets except SRR5414541 and SRR5221560, there was no pattern for the position on reads for the first k-mer marked as suspect but in these two read sets, over 70% of the reads trimmed were clipped at the start of the read.

## Conclusions

Sequence assembly of reads for microbial genomes for applications such as real-time pathogen detection in foodborne and clinical samples require high sequence quality, sufficient contiguity, and good scaling in performance with compute resources. Reproducibility of the results is also a critical requirement in production systems handling large volumes of data, especially for public health applications. We presented a de-novo assembler, SKESA, that does strategic k-mer extension for scrupulous assemblies and achieves desired properties for the assembly of reads from the microbial genomes sequenced using Illumina sequencing platform. The assembly approach utilizes DeBruijn graphs and conservative heuristics using k-mer counts of alternate choices to decide between extending or creating a break in the assembly. Multiple iterations with several k-mer sizes up to the expected length of insert size for paired reads are used to produce the assembly. SKESA also handles presence of low-level contamination from different samples gracefully.

We compared SKESA to two widely used de-novo assemblers: SPAdes, a versatile assembler in the range of sequences it can assemble, and MegaHit, a very fast assembler. For the specific application of microbial assemblies SKESA was designed for, we showed that the quality of SKESA assemblies is better than both SPAdes and MegaHit, and its speed is comparable to MegaHit. Contiguity of SKESA and MegaHit drop with increasing level of contamination while SPAdes maintains contiguity. The same assembly is produced by SKESA on the same input when runs are performed multiple times or when compute resources provided to the runs are changed. The same does not hold true for SPAdes and MegaHit.

Future work for SKESA includes (i) using a k-mer histogram to make a quick assessment of whether contamination in the sample is high enough to warrant no assembly, (ii) exploring extensions to other sequencing technologies such as nanopore that have good genome coverage but suffer from high error rate, (iii) exploring extension to diploid genomes with heterozygous sites assembled using appropriate ambiguity code, (iv) understanding behavior on large genomes, and (v) adding modules to detect rare cases where read trimming removes k-mers that can be self-assembled.

In all future work, our goal will continue to be to produce assemblies with close to perfect base level accuracy.

## Methods

We present the algorithm design for SKESA, some important implementation details, design of test sets used for running time and assembly quality comparisons, and command lines used for doing the runs. We compare SKESA to SPAdes v3.11.1 and MegaHit v1.1.2. Assessment of assembly quality was done using QUAST. We attempted to use misFinder [45] and ReMILO [46] but neither worked reliably. When misFinder or ReMILO worked, results were similar to that of QUAST.

### Algorithm design for SKESA

**Fig. 4:**
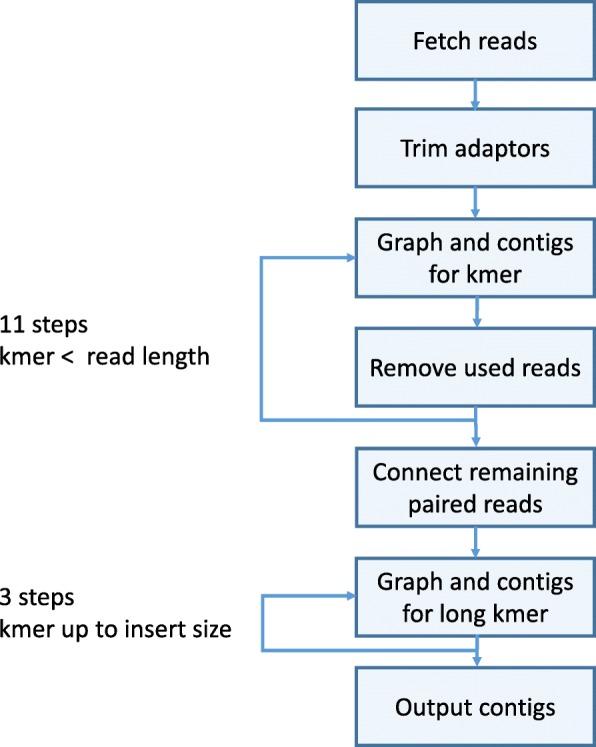
SKESA flowchart: flowchart describing main steps in the algorithm used by SKESA for assembly
- Detection of parameters: A user should specify the option for whether the reads are paired or single and the compute resources available. All other parameters are determined internally by SKESA unless explicitly specified.
- Assembly using a specific k-mer size: In each iteration, the assembly process uses the DeBruijn graph for that k-mer size and an empty or current set of contigs. Multiple k-mer sizes are used. Short k-mers can assemble low-coverage areas of the genome while long k-mers can resolve repeats.
- Marking reads: This module decides reads that are *used up* and no longer needed for future iterations.

A flowchart describing the main modules of SKESA is shown in Fig. 4. Other than reading input and writing output, the four main parts of the SKESA algorithm are as follows:

- Trimming of reads.

After trimming of reads, the rest of the SKESA process uses trimmed reads only and we overload “read” to mean trimmed reads after this step. If input has paired reads, after iterating using k-mers up to mate length, any read still available for assembly has a mini-assembly performed treating its mates as ends of contigs. Assembled reads are used for generating three sets of k-mers that are longer than the mate size and up to the expected insert size. No explicit error correction of reads is done by SKESA as the heuristics of SKESA can handle the errors in a typical illumina read set. Next, we describe each of the five modules.

#### Read trimming

K-mer size of 19 is used for counting frequency of k-mers in the read set. If a k-mer is seen in at least *V*_*f*_ fraction of reads (default 0.05), it is considered *suspect* and used for trimming reads. Starting from the first k-mer in a mate and checking all consecutive k-mers, the first occurrence of a k-mer flagged as suspect trims the rest of the mate.

#### Parameter detection

**Fig. 5:**
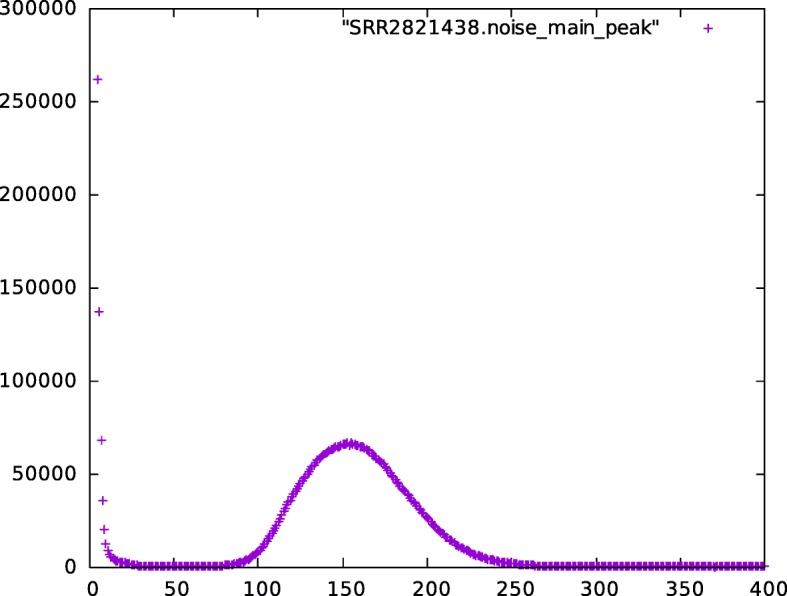
Main distribution in SRR2821438: histogram for frequency of 21-mers seen in SRR2821438 with counts on *X* axis up to 400 and number of 21-mers with that count on *Y* axis

**Fig. 6:**
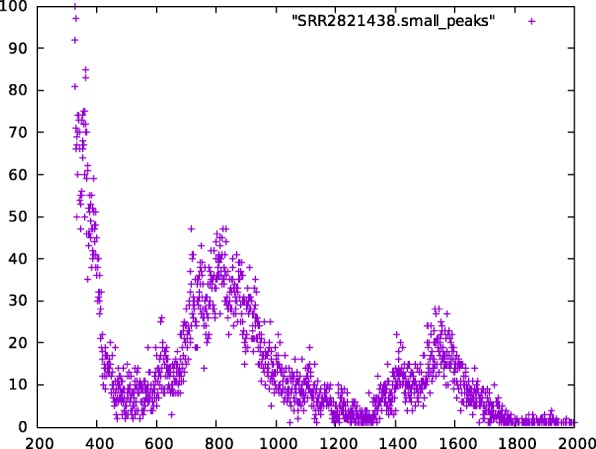
Small distributions in SRR2821438: histogram for frequency of 21-mers seen in SRR2821438 with counts on *X* axis between 325 and 2000 and number of 21-mers with that count on *Y* axis

SKESA builds a histogram for frequency of k-mers at the minimal k-mer length *K*_*min*_ (default 21) seen in trimmed reads. Using the histogram, it decides the peak where the distribution around the peak likely corresponds to the k-mers from the genome being assembled. This distribution is used to estimate the genome size *G*. If no peak is detected, then 80% of the entire distribution is used as an estimate of *G*. Additional peaks present and distributions around those peaks are usually due to noise, repeats, or plasmids. For example, Figs. 5 and 6 are two parts of the histogram for SRR2821438 generated with 21-mers. Figure 5 shows the noise and distribution for k-mers from the genome and Fig. 6 shows two peaks that have much higher k-mer counts but relatively few k-mers as compared to the distributions in Fig. 5.

To account for more noise in high-coverage read sets, the minimum frequency count, *C*_*min*_ is computed as *max*(2,*T*/(*G*∗50)) where *T* is the total length of reads. All k-mers with count below the minimum count are ignored in the assembly. The program also computes *C*_*max*_ as *max*(10,*T*/(*G*∗10)). Choice of k-mer lengths is made by SKESA using *K*_*min*_, number of steps *S* (default 11), and maximal k-mer length *K*_*max*_ where *K*_*max*_ is determined using the average of all mate lengths *A*_*read*_ and counts of k-mers. *K*_*max*_ is initially set to *A*_*read*_. If the average count of k-mers at current *K*_*max*_ is below the desired count *C*_*max*_, then *K*_*max*_ is iteratively reduced by *A*_*read*_/25 bases until a *K*_*max*_ with average count of at least *C*_*max*_ is found. If *K*_*max*_ is more than 1.5 times *K*_*min*_, then *S*−2 additional k-mers between *K*_*min*_ and *K*_*max*_ are chosen. These are odd integers that are spread evenly. Otherwise, only *K*_*min*_ is used for an assembly and a warning that iterations are disabled is printed.

For paired runs, if the insert size *I* is not provided, then it is estimated using a random sample of 10,000 reads. An unambiguous assembly for each of these reads with the two mates as ends of contigs is attempted using *K*_*min*_. Using the length of reads assembled, insert size *I* is estimated. Three additional k-mer sizes added for additional iterations are 1.25*K*_*max*_, (1.25*K*_*max*_+*I*)/2 and *I*. The program also uses 3*I* as the *maximal insert size* expected for any read.

#### Assembling using a specific k-mer size *K*

All k-mers of length *K* with frequency at least *C*_*min*_ are generated. If a main peak in the histogram of the frequency of generated k-mers is detected, the left low end of the distribution around the main peak is called the *Valley* for the iteration and only k-mers with count above the valley are used to start new contigs. A valley is set to zero if no main peak is found in the histogram.

At any stage, an attempt to extend an end of a contig by the next base results in three possibilities: (i) no k-mer extension is possible, (ii) only one k-mer extension is possible, or (iii) there are alternate choices. In the first case, the end of the contig has been reached and no further extension is possible. In the second case, the contig is extended by one base only if the extension from the new k-mer produced by addition of the base to the previous k-mer (last k-mer of the end of the contig) is also possible using the same criteria used for extending from previous k-mer to the new k-mer. In the third case, all choices with counts below the threshold for extension (default 0.1) with respect to the maximum counts for any choice are considered as noise and dropped. If more than one choice for extension survives this count based filtering, potential Illumina strand-specific systematic error signatures are evaluated. The program does this by comparing counts observed on both strands. If there is a choice with counts balanced on both strands, all choices with counts seen in predominately one strand are dropped. If more than one choice for extension survives this strand-based filtering, each choice is used for finding paths that are extended by a maximum of *max*(100,*K*) steps. If only one path survives, then it is kept while others are removed as dead ends. If more than one path survives, a contig break is created. When a contig reaches the stage where an extension is no longer possible, the last k-mer bases are removed to ensure that the sequence built was verified by assembling from both directions.

In the process of contig extension, suppose contig *C* is being extended by a base *b* resulting in last k-mer *L* in *C* that includes *b*. The program checks if *L* is already present in any contig *D*. If no such *D* exists, *C* is extended using *L*. If such a *D* exists and *L* is at the end of *D*, program merges *C* and *D*. Otherwise, *C* is not extended. This ensures that no k-mer is included more than once in the assembled contigs in the iteration for that k-mer.

#### Marking reads as used

After each iteration, the reads that have a k-mer located deeper than *buffer zoneM* inside a contig are marked as *used* as they cannot contribute any new information. Value of *M* is *I*+50+*F* where flank *F* is set to *K*_*max*_ if reads being removed are the input reads and not the ones assembled as a pair for generating k-mers larger than the mate size. Otherwise, *F* is set to zero.

#### Connecting paired reads

If input is for paired reads, after the iterations using k-mers up to mate length, the program attempts to unambiguously connect reads that are not marked as used. Starting from the last k-mer of the first mate to first k-mer of the second mate, all paths up to maximal insert size are assembled. Similarly, an assembly from reverse complement of the first k-mer of the second mate to reverse complement of the last k-mer of the first mate is attempted. If both produce only one path and sequence is same for both paths (except for reverse complement), the assembled sequence is used for generating longer k-mers. For pairs that are inside the buffer zone *M*, sequence from the contig is used for generating long k-mers.

### Implementation

#### Dependencies

SKESA uses the freely available Boost library [47]. If direct access to SRA is desired for retrieving reads, then the SRA toolkit library is also needed. For k-mers, the long integer implementation from [48] is used and is included in the SKESA package.

#### K-mer counting and searching

For k-mer counting, two options are implemented. By default, all k-mers from all reads are generated and counted after sorting. If the memory available is not sufficient to store k-mers from all reads, then a hash function is used to determine smaller batches of k-mers to process from all reads in several rounds. In each round, k-mers that do not meet the threshold *C*_*min*_ are discarded. The second method uses a hash table and a Bloom filter [49] to filter out k-mers that have counts below the threshold *C*_*min*_. A small number of k-mers below the *C*_*min*_ threshold not detected by the Bloom filter are removed later. Generally, the method utilizing the Bloom filter consumes less memory during the counting but the resulting hash table is larger than the default method that uses a sorted array for counting.

For k-mer searching, binary search is used for finding k-mers when they are stored in a sorted array. In the second implementation that uses a hash table, k-mer search uses the hash function to directly find the index in the hash table.

#### Multi-threading

All steps in the SKESA implementation are highly multi-threaded. No intermediate or temporary output is generated in order to reduce the load on storage bandwidth when runs are done with many compute nodes available.

For counting k-mers using sorting, a hash function is used to separate generated k-mers into non-overlapping bins. Each bin is sorted and counted by a separate thread. The sorted and counted bins are merged afterwards. Both the Bloom filter and hash table are implemented as lock-free structures using compare-and-swap (CAS) hardware operation.

The assembly process is designed not to include the same k-mer in different contigs in the iteration for that k-mer size. To accomplish this, each k-mer in the DeBruijn graph has a lock-free atomic variable. When a k-mer is used in a contig, this variable is set and that prevents any further use of the k-mer.

During a multi-threaded operation, several threads could start assembling the same contig from different starting k-mers. However, at some point, they will collide on a k-mer that will stop further assembly, resulting in contig fragments. After each iteration, all assembled sequences are analyzed and connected to each other appropriately to account for this collision. If a contig connects to itself, this is recognized and contig is marked as circular.

Multi-threading results in random orientation of contigs. Circular contigs also have random breakpoints. After each iteration, only the contig or its reverse complement is kept depending on which one starts with the smaller k-mer in the lexicographic order. Each circular contig and its reverse complement are checked for the smallest k-mer and that k-mer is chosen as the breakpoint. All contigs are then sorted. These steps guarantee that each iteration starts from the same state regardless of the number of cores and memory used for the assembly.

#### Output

Alphabetically sorted assembled contigs are output as a FASTA file. Each contig is named with format Contig_N_C where N is a sequential contig number starting at one and C is the average of count for k-mers in the contig at k-mer size *K*_*min*_. If a contig was recognized as circular, contig name is suffixed by _Circ.

### Test sets and testing criteria for comparison

Five microbial test sets were used for comparing assemblers: *run time set* covering a range of microbial species, *benchmark set* where a reference assembly and reads for the same sample are available, *random set* of read sets from SRA for four microbial species, *contamination set* where contamination is spiked in at different levels, and *substrings set* where all substrings of a genome at various lengths were used as input reads. We used QUAST for computing the number of misassemblies and mismatches per hundred kilobases of the reference assembly. Assembly contiguity was assessed using N50 criteria. Assembly length discrepancy was assessed as *L*_*R*_+*L*_*A*_−2∗*C*_*RA*_ where *L*_*R*_ is the length of reference assembly, *L*_*A*_ is the length of assembly being tested, and *C*_*RA*_ is the length reported as aligned between *A* and *R* by QUAST. The composition of test sets [41] and their use for various testing criteria are described next.

#### Run time set

The run time set shown in Table 6 consists of 56 read sets representing 34 microbial species. This set was selected by the PDP team from FDA-ARGOS available in May 2016 and publications. For running time, each read set was run three times on three different settings of number of cores and memory. Settings used were 4 cores and 16 Gb, 8 cores and 32 Gb, and 12 cores and 32 Gb. Runs were done on CentoS 7.

**Table 6:** Runs and species for testing running time performance

SRA run	Species
SRR2820668	*Achromobacter xylosoxidans*
SRR2822445	*Achromobacter xylosoxidans*
SRR2821368	*Achromobacter xylosoxidans*
SRR2821369	*Achromobacter xylosoxidans*
SRR2823707	*Bartonella bacilliformis*
SRR2823715	*Bordetella bronchiseptica*
SRR2823716	*Bordetella bronchiseptica*
SRR2824043	*Bordetella pertussis*
SRR2822462	*Citrobacter amalonaticus*
SRR2818794	*Citrobacter amalonaticus*
SRR1284629	*Citrobacter freundii*
SRR2821773	*Citrobacter* sp.
SRR1515967	*Enterobacter cloacae*
SRR1576778	*Enterobacter cloacae* complex
SRR1576808	*Enterobacter cloacae* complex
SRR2822449	*Enterococcus* sp.
ERR008613	*Escherichia coli*
ERR022075	*Escherichia coli*
SRR530851	*Escherichia coli*
SRR587217	*Escherichia coli*
SRR2817810	*Grimontia hollisae*
SRR2817811	*Grimontia hollisae*
SRR2822309	*Hafnia alvei*
ERR351267	*Helicobacter pylori*
SRR2821438	*Klebsiella aerogenes*
SRR2820617	*Klebsiella aerogenes*
SRR2820618	*Klebsiella aerogenes*
SRR1501122	*Klebsiella oxytoca*
SRR1427234	*Klebsiella pneumoniae*
SRR1505904	*Klebsiella pneumoniae*
SRR1427243	*Klebsiella pneumoniae*
SRR1501128	*Klebsiella pneumoniae*
SRR1510963	*Klebsiella pneumoniae*
SRR941212	*Mannheimia haemolytica*
SRR2823701	*Morganella morganii*
SRR2822442	*Pantoea agglomerans*
SRR2820663	*Providencia stuartii*
SRR498276	*Salmonella enterica*
SRR2814419	*Salmonella enterica*
SRR2814420	*Salmonella enterica*
SRR2819198	*Serratia liquefaciens*
SRR2812569	*Shigella sonnei*
SRR2812570	*Shigella sonnei*
SRR1206476	*Staphylococcus aureus*
SRR2822404	*Staphylococcus aureus*
SRR2820641	*Staphylococcus lugdunensis*
SRR2820657	*Staphylococcus lugdunensis*
SRR2822469	*Staphylococcus saprophyticus*
SRR2820294	*Staphylococcus saprophyticus*
SRR2819094	*Staphylococcus simulans*
SRR2820674	*Streptococcus pyogenes*
SRR2815879	*Vibrio fluvialis*
SRR2817447	*Vibrio harveyi*
SRR2818033	*Vibrio mimicus*
SRR2818092	*Vibrio parahaemolyticus*
SRR2818127	*Vibrio vulnificus*

#### Benchmark set

FDA-ARGOS had 403 read sets with reads sequenced using Illumina and a good quality assembly in GenBank in March 2018. Of these, SPAdes failed to produce an assembly for 18 read sets. For four read sets (SRR2814770, SRR2820671, SRR5413268, and SRR5866647), QUAST reported more than 10 mismatches per 100 Kb for all assembly methods. We show quality assessment results using the remaining 381 read sets.

#### Random set

Four of the most common foodborne pathogen species are *Salmonella Enterica, Listeria Monocytogenes, Escherichia coli* and *Shigella*, and *Campylobacter*. From SRA, we randomly selected 5500 read sets from these species sequenced on Illumina machines, sorted them by number of bases in reads, and dropped 250 runs each with lowest and highest base counts. The remaining 5000 reads sets used as the random set have 3306 Salmonella, 428 Listeria, 773 Escherichia, 148 Shigella, and 345 Campylobacter. These sets were used to test the contiguity of assemblies. Runs for the random set were done in an uncontrolled environment on compute farm. We note that the CPU times reported by the compute farm (data not shown) on these 5000 read sets corroborate the run time performance presented in Table 1.

#### Contamination set

Paired reads were generated from *Salmonella typhimurium* strain LT2 (NC_003197.1) randomly covering the genome at 60x. For adding contamination, the same reference genome was randomly mutated at 0.1% of the positions. Reads from the mutated genome at coverage of 3 ×, 6 ×, 9 ×, 12 ×, and 15 × were added to the clean set to generate six simulated sets for testing the effect of contamination ranging from no contamination to a fifth of the reads coming from the mutated reference. All reads generated had mates that were 150 bp in length and insert size of 300 bp. These sets were used for assessing sequence quality and behavior of contiguity at different levels of contamination.

#### Substrings set

Single reads were generated from Salmonella Typhimurium strain LT2 (NC_003197.1) where for each value of *K* from 22 to 152, all substrings of that length were used as input reads for assembly. One read per base pair of the genome was generated resulting in coverage of *K* for a read set generated with substring length of *K*. Substrings generated at even positions of the reference genome were reverse complemented. As such, this test varied length and coverage but did not introduce any errors. These sets were used for assessing sequence quality and behavior of contiguity at different levels of coverage and read length.

### Commands for programs

For doing the runs comparing performance of different software, defaults were used except for parameters that specify the number of cores and memory allowed. For SKESA, the flag for specifying that reads are paired was also given as appropriate. Command lines for, say, running SRR498276 for SKESA, SPAdes, and MegaHit are as follows:


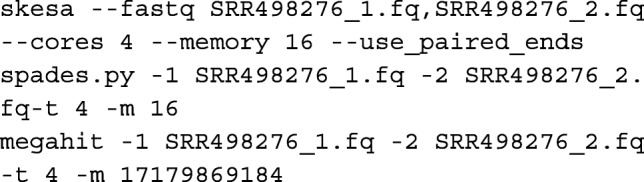


For SKESA, if direct SRA access is available, one can instead do the following:


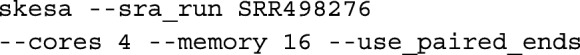


For the substring set that has single reads, option use_paired_ends is not specified for SKESA runs and option only_assembler is specified for SPAdes runs.

For SKESA, we recommend providing 16 Gb of memory and using defaults so it can internally tune the parameters for best results. Additional options are exposed for users who may wish to use SKESA for non-standard applications or understand SKESA behavior.

## Availability and requirements

Project name: SKESA

Source code: https://github.com/ncbi/SKESA/releases

Archived version: http://doi.org/10.5281/zenodo.1407162

Operating system: Linux

Other requirements: BOOST

License: Freely available to the public for use with exception of bundled third party code. The third party code contained in SKESA release is available under GNU GPLv3. See https://github.com/ncbi/SKESA/blob/master/LICENSE for details.

## Additional file

Additional file 1 Supplementary notes, tables, and figures. (PDF 166 KB)

## References

[1] Lugli Gabriele Andrea, Milani Christian, Mancabelli Leonardo, van Sinderen Douwe, Ventura Marco (2016). MEGAnnotator: a user-friendly pipeline for microbial genomes assembly and annotation. FEMS Microbiology Letters.

[2] Pina-Martins F, Vieira BM, Seabra SG, Batista D, Paulo OS. 4pipe4–a 454 data analysis pipeline for SNP detection in datasets with no reference sequence or strain information. BMC Bioinformatics. 2016; 17:41. 10.1186/s12859-016-0892-1. 10.1186/s12859-016-0892-1 PMC4719533 26787189

[3] Lai B, Wang F, Wang X, Duan L, Zhu H. Intemap: integrated metagenomic assembly pipeline for NGS short reads. BMC Bioinformatics. 2015; 16:244. 10.1186/s12859-015-0686-x. 10.1186/s12859-015-0686-x PMC4545859 26250558

[4] Wolfinger Michael T., Fallmann Jörg, Eggenhofer Florian, Amman Fabian (2015). ViennaNGS: A toolbox for building efficient next- generation sequencing analysis pipelines. F1000Research.

[5] Tritt Andrew, Eisen Jonathan A., Facciotti Marc T., Darling Aaron E. (2012). An Integrated Pipeline for de Novo Assembly of Microbial Genomes. PLoS ONE.

[6] Xiao Wenming, Wu Leihong, Yavas Gokhan, Simonyan Vahan, Ning Baitang, Hong Huixiao (2016). Challenges, Solutions, and Quality Metrics of Personal Genome Assembly in Advancing Precision Medicine. Pharmaceutics.

[7] About GMI - Vision and Objectives. http://www.globalmicrobialidentifier.org/about-gmi/vision-and-objectives.

[8] Allard MW, Strain E, Melka D, Bunning K, Musser SM (2016). Practical value of food pathogen traceability through building a whole-genome sequencing network and database. J Clin Microbiol.

[9] den Bakker HC, Allard MW, Bopp D, Brown EW, Fontana J (2014). Rapid whole-genome sequencing for surveillance of Salmonella enterica serovar Enteritidis. Emerg Infect Dis.

[10] Snitkin E. S., Zelazny A. M., Thomas P. J., Stock F., Henderson D. K., Palmore T. N., Segre J. A. (2012). Tracking a Hospital Outbreak of Carbapenem-Resistant Klebsiella pneumoniae with Whole-Genome Sequencing. Science Translational Medicine.

[11] Jackson BR, Tarr C, Strain E, Jackson KA, Conrad A (2016). Implementation of nationwide real-time whole-genome sequencing to enhance listeriosis outbreak detection and investigation. Clin Infect Dis.

[12] van Duin D, Perez F, Rudin SD, Cober E, Hanrahan J (2014). Surveillance of carbapenem-resistant Klebsiella pneumoniae: tracking molecular epidemiology and outcomes through a regional network. Antimicrob Agents Chemother.

[13] Katz LS, Griswold T, Williams-Newkirk AJ, Wagner D, Petkau A, et al.A comparative analysis of the lyve-set phylogenomics pipeline for genomic epidemiology of foodborne pathogens. Front Microbiol. 2017;8. 10.3389/fmicb.2017.00375. 10.3389/fmicb.2017.00375 PMC5346554 28348549

[14] Lüth S, Sylvia K, Sascha AD (2018). Whole genome sequencing as a typing tool for foodborne pathogens like Listeria monocytogenes – the way towards global harmonisation and data exchange. Trends Food Sci Technol.

[15] Sekse C, Holst-Jensen A, Dobrindt U, Johannessen GS, Li W, Spilsberg B, Shi J. High throughput sequencing for detection of foodborne pathogens. Front Microbiol. 2017;8. 10.3389/fmicb.2017.02029. 10.3389/fmicb.2017.02029 PMC5655695 29104564

[16] Allard MW, Bell R, Ferreira CM, Gonzalez-Escalona N, Hoffmann M (2018). Genomics of foodborne pathogens for microbial food safety. Curr Opin Biotechnol.

[17] Meacham Frazer, Boffelli Dario, Dhahbi Joseph, Martin David IK, Singer Meromit, Pachter Lior (2011). Identification and correction of systematic error in high-throughput sequence data. BMC Bioinformatics.

[18] Laehnemann D, Borkhardt A, McHardy AC (2016). Denoising DNA deep sequencing data-high-throughput sequencing errors and their correction. Brief Bioinform.

[19] Infectious Disease Next Generation Sequencing Based Diagnostic Devices: Microbial identification and detection of antimicrobial resistance and virulence markers. https://www.fda.gov/downloads/MedicalDevices/DeviceRegulationandGuidance/GuidanceDocuments/UCM500441.pdf.

[20] MiSeqⓇ System Guide. https://support.illumina.com/content/dam/illumina-support/documents/documentation/system_documentation/miseq/miseq-system-guide-for-local-run-manager-15027617-04.pdf.

[21] Luo R, Liu B, Xie Y, Li Z, Huang W, et al. Soapdenovo2: an empirically improved memory-efficient short-read de novo assembler. Gigascience. 2012; 1(1):18. 10.1186/2047-217X-1-18. 10.1186/2047-217X-1-18 PMC3626529 23587118

[22] Zerbino D. R., Birney E. (2008). Velvet: Algorithms for de novo short read assembly using de Bruijn graphs. Genome Research.

[23] Zimin Aleksey V., Marçais Guillaume, Puiu Daniela, Roberts Michael, Salzberg Steven L., Yorke James A. (2013). The MaSuRCA genome assembler. Bioinformatics.

[24] Bankevich Anton, Nurk Sergey, Antipov Dmitry, Gurevich Alexey A., Dvorkin Mikhail, Kulikov Alexander S., Lesin Valery M., Nikolenko Sergey I., Pham Son, Prjibelski Andrey D., Pyshkin Alexey V., Sirotkin Alexander V., Vyahhi Nikolay, Tesler Glenn, Alekseyev Max A., Pevzner Pavel A. (2012). SPAdes: A New Genome Assembly Algorithm and Its Applications to Single-Cell Sequencing. Journal of Computational Biology.

[25] Maccallum I, Przybylski D, Gnerre S, Burton J, Shlyakhter I (2009). Allpaths 2: small genomes assembled accurately and with high continuity from short paired reads. Genome Biol.

[26] Sommer DD, Delcher AL, Salzberg SL, Pop M. Minimus: a fast, lightweight genome assembler. BMC Bioinformatics. 2007; 8:64. 10.1186/1471-2105-m. 10.1186/1471-2105-8-64 PMC1821043 17324286

[27] Treangen Todd J, Koren Sergey, Sommer Daniel D, Liu Bo, Astrovskaya Irina, Ondov Brian, Darling Aaron E, Phillippy Adam M, Pop Mihai (2013). MetAMOS: a modular and open source metagenomic assembly and analysis pipeline. Genome Biology.

[28] Simpson JT, Wong K, Jackman SD (2009). Abyss: a parallel assembler for short read sequence data. Genome Res.

[29] Safonova Yana, Bankevich Anton, Pevzner Pavel A. (2015). dipSPAdes: Assembler for Highly Polymorphic Diploid Genomes. Journal of Computational Biology.

[30] Kultima Jens Roat, Coelho Luis Pedro, Forslund Kristoffer, Huerta-Cepas Jaime, Li Simone S., Driessen Marja, Voigt Anita Yvonne, Zeller Georg, Sunagawa Shinichi, Bork Peer (2016). MOCAT2: a metagenomic assembly, annotation and profiling framework. Bioinformatics.

[31] Guo Xuan, Yu Ning, Ding Xiaojun, Wang Jianxin, Pan Yi (2015). DIME: A Novel Framework for De Novo Metagenomic Sequence Assembly. Journal of Computational Biology.

[32] Peng Y., Leung H. C. M., Yiu S. M., Chin F. Y. L. (2011). Meta-IDBA: a de Novo assembler for metagenomic data. Bioinformatics.

[33] Afiahayati, Sato K., Sakakibara Y. (2014). MetaVelvet-SL: an extension of the Velvet assembler to a de novo metagenomic assembler utilizing supervised learning. DNA Research.

[34] Haider Bahlul, Ahn Tae-Hyuk, Bushnell Brian, Chai Juanjuan, Copeland Alex, Pan Chongle (2014). Omega: an Overlap-graph de novo Assembler for Metagenomics. Bioinformatics.

[35] Li Dinghua, Luo Ruibang, Liu Chi-Man, Leung Chi-Ming, Ting Hing-Fung, Sadakane Kunihiko, Yamashita Hiroshi, Lam Tak-Wah (2016). MEGAHIT v1.0: A fast and scalable metagenome assembler driven by advanced methodologies and community practices. Methods.

[36] Peng Y., Leung H. C. M., Yiu S. M., Chin F. Y. L. (2012). IDBA-UD: a de novo assembler for single-cell and metagenomic sequencing data with highly uneven depth. Bioinformatics.

[37] Shelton JM, Coleman MC, Herndon N, Lu N, Lam ET, et al. Tools and pipelines for bionano data: molecule assembly pipeline and fasta super scaffolding tool. BMC Genomics. 2015; 16(1):734. 10.1186/s12864-015-1911-8. 10.1186/s12864-015-1911-8 PMC4587741 26416786

[38] Wences AH, Schatz MC. Metassembler: merging and optimizing de novo genome assemblies. Genome Biol. 2015; 16:207. 10.1186/s13059-015-0764-4. 10.1186/s13059-015-0764-4 PMC4581417 26403281

[39] Steinberg KM, Schneider VA, Graves-Lindsay TA, Fulton RS, Agarwala R (2014). Single haplotype assembly of the human genome from a hydatidiform mole. Genome Res.

[40] Gnerre S, Maccallum I, Przybylski D, Ribeiro FJ, Burton JN (2011). High-quality draft assemblies of mammalian genomes from massively parallel sequence data. Proc Natl Acad Sci USA.

[41] Souvorov A, Agarwala R, DJ L.SKESA Data. http://ftp.ncbi.nlm.nih.gov/pub/agarwala/skesa/datasets.

[42] Gurevich Alexey, Saveliev Vladislav, Vyahhi Nikolay, Tesler Glenn (2013). QUAST: quality assessment tool for genome assemblies. Bioinformatics.

[43] Souvorov A, Agarwala R, DJ L.SKESA Source Code; GitHub 2018. https://github.com/ncbi/SKESA/releases.

[44] Souvorov A, Agarwala R, DJ L.SKESA Source Code; Zenodo 2018. https://zenodo.org/record/1407162.

[45] Zhu X, Leung HCM, Wang R, Chin FYL, Yiu SM, et al. Misfinder: identify mis-assemblies in an unbiased manner using reference and paired-end reads. BMC Bioinformatics. 2015; 16:386. 10.1186/s12859-015-0818-3. 10.1186/s12859-015-0818-3 PMC4647709 26573684

[46] Bao E, Song C, L L. (2018). Remilo: reference assisted misassembly detection algorithm using short and long reads. Bioinformatics.

[47] BOOST C++ Libraries. https://www.boost.org/.

[48] Drezen E, Rizk G, Chikhi R, Deltel C, Lemaitre C (2014). Gatb: Genome assembly & analysis tool box. Bioinformatics.

[49] Putze F, Sanders P, Singler J. Cache-, hash-, and space-efficient bloom filters. J Exp Algorithmics. 2009;14. https://dl.acm.org/citation.cfm?doid=1498698.1594230.

