# Supplementary material for: SKESA: strategic k-mer extension for scrupulous assemblies
Source: Genome Biol. 2018 Oct 4;19:153. doi: 10.1186/s13059-018-1540-z (PMC6172800; doi:10.1186/s13059-018-1540-z)
Supplement: Supplementary file 1 — Supplementary notes, tables, and figures. (PDF 166 KB) [file 13059_2018_1540_MOESM1_ESM.pdf]

Supplementary material for
SKESA: strategic k-mer extension for scrupulous assemblies

Alexandre Souvorov Richa Agarwala David J. Lipman

Command lines for IDBA and ABySS runs

Defaults were used for IDBA and ABySS except for the number of cores and parameters needed to specify or limit size of k-mers. Command lines for, say, running SRR498276 for IDBA and ABySS using 4 cores are as follows:

```
idba_ud -r SRR498276.reads.fa -o temp_idba --num_threads 4
abyss-pe name=SRR498276 k=64 in=../SRR498276.reads.fa -C temp_abyss -j 4
```

For running IDBA on substrings test set, parameter `--maxk` was set to the length of substrings for runs that had reads with length at most 100 bp and to 100 for the rest. For running ABySS on substrings test set, parameter k was set to 21 for runs with read lengths in range 22 to 42, k was set to 32 for runs with read lengths in range 43 to 96, and k was set to 48 for runs with read lengths longer than 96 bases. Command lines for, say, running substrings with read length 90 for IDBA and ABySS using a single core were:

```
idba_ud -l reads.96.fa -o temp_idba_96 --num_threads 1 --maxk 96
abyss-pe name=reads.96 k=32 se=../reads.96.fa -C temp_abyss_96 -j 1
```

Tables and Figures with IDBA and ABySS results added

We do not report run time comparison table in the supplementary material as one cannot control memory usage for IDBA and ABySS. In general, time taken by IDBA and ABySS is of the same order as that of SPAdes. Tables 2 through 5 and Figures 1 through 3 of the main manuscript with additional information for IDBA and ABySS included are given below where for each of these tables and figures, the corresponding table or figure number is prefixed by letter 'S'.

Table S2: Number of misassemblies in 381 inputs in the benchmark set.

Count	SKESA	SPAdes	MegaHit	IDBA	ABySS
0	214	172	128	224	250
1	83	98	91	76	79
2	40	43	66	32	26
3	13	30	30	8	6
4	9	12	18	11	9
5	7	7	15	5	8
6	2	3	10	7	0
7	2	0	5	1	1
8	1	1	3	2	0
9	0	0	2	2	0
10+	10	15	13	13	2
Median	0	1	1	0	0

Table S3: Mismatches per 100 Kb as reported by QUAST for benchmark and contamination sets.

Benchmark set					
Measure	SKESA	SPAdes	MegaHit	IDBA	ABySS
Median	0.08	2.76	1.89	0.45	3.79
Maximum	7.78	41.60	31.94	26.56	29.40
Average	0.40	3.21	2.79	0.98	5.17
Assembly counts in benchmark set					
Mismatches range	SKESA	SPAdes	MegaHit	IDBA	ABySS
0	105	1	1	13	3
0.01 – 1	247	40	80	267	34
1.01 – 2	9	76	121	53	58
2.01 – 3	9	89	58	22	51
3.01 – 4	1	71	45	11	49
> 4	10	104	76	15	186
Mismatches reported in contamination set					
Set	SKESA	SPAdes	MegaHit	IDBA	ABySS
No contamination	0	1.44	3.83	1.50	5.72
3x contamination	0	1.42	3.21	1.50	5.72
6x contamination	0	1.44	3.02	1.40	5.57
9x contamination	0.02	1.61	4.38	1.40	5.46
12x contamination	0.02	1.52	4.96	1.11	3.77
15x contamination	0.04	1.50	5.83	1.36	3.34

Table S4: Deviation of assembly length produced by the assemblers from the assembly length of the reference as computed using aligned length reported by QUAST and assembly lengths for benchmark and contamination sets.

Benchmark set					
Measure	SKESA	SPAdes	MegaHit	IDBA	ABySS
Median	2.72	10.91	5.59	5.99	2.98
Maximum	135.75	775.14	407.78	433.23	144.61
Average	4.61	57.98	24.23	26.81	4.91
Deviation in contamination set					
Contamination	SKESA	SPAdes	MegaHit	IDBA	ABySS
None	1.33	1.68	1.35	1.54	0.94
3x	1.36	1.68	1.33	1.70	0.93
6x	1.33	1.68	1.30	1.68	0.96
9x	1.36	1.67	1.47	1.67	1.32
12x	1.41	1.68	2.05	1.71	1.63
15x	1.44	1.68	2.96	1.71	1.86

Table S5: Contiguity for benchmark, random, and contamination sets.

Benchmark set					
N50 measure	SKESA	SPAdes	MegaHit	IDBA	ABySS
<= 10 Kb	14	69	19	20	12
10001 – 50 Kb	40	41	46	67	43
50001 – 100 Kb	41	56	67	100	111
100001 – 250 Kb	191	169	197	154	185
250001 – 500 Kb	77	43	48	38	26
> 500 Kb	18	3	4	2	4
Median	170647	117340	124833	101678	107730
Minimum	1832	364	687	659	2104
Maximum	1197860	622367	617087	617493	549807
Average	195141	131823	146706	119363	125993
N50 statistic in contamination set					
Contamination	SKESA	SPAdes	MegaHit	IDBA	ABySS
None	282763	260531	202384	149607	220096
3x	282763	260531	202384	149607	220096
6x	282763	260532	202384	149607	220096
9x	225630	260531	151916	149607	183921
12x	77455	260531	107175	149607	180813
15x	42440	260531	65124	149607	180813
Random set					
N50 measure	SKESA	SPAdes	MegaHit	IDBA	ABySS
<= 10 Kb	6	10	6	389	21
10001 – 50 Kb	349	206	285	2140	1051
50001 – 100 Kb	788	409	1516	942	1730
100001 – 250 Kb	2307	2369	2889	1420	2021
250001 – 500 Kb	1324	1616	266	82	166
> 500 Kb	226	390	38	27	11
Median	170877	208907	117074	48953	91918.5
Minimum	2414	209	4182	301	1747
Maximum	1545488	1530182	1499532	1499368	1502369
Average	213847	255079	136339	77447.3	103982

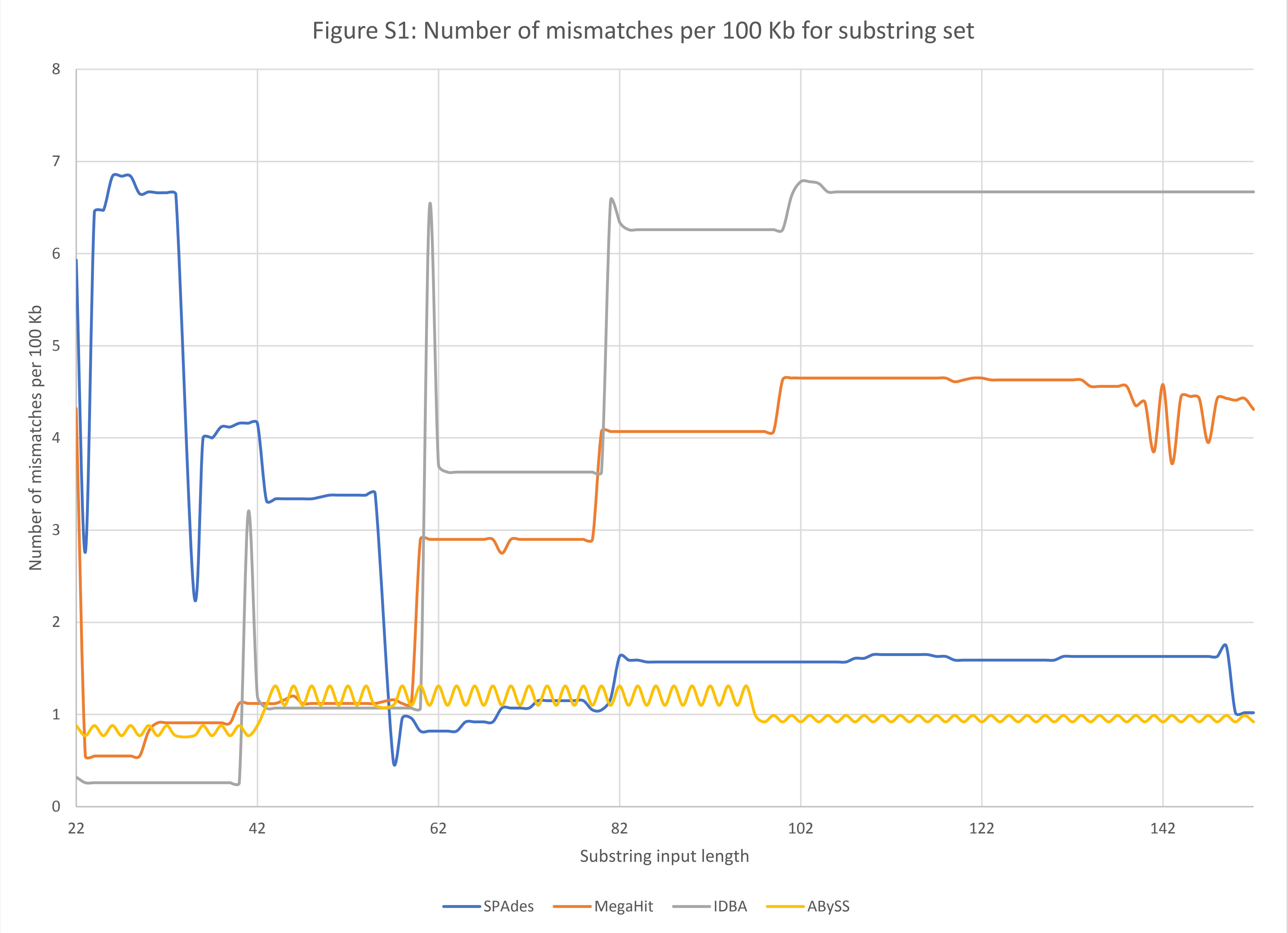
Figure S1: Number of mismatches per 100 Kb for substring set
Number of mismatches per 100 Kb
0
1
2
3
4
5
6
7
8
22
42
62
82
102
122
142
Substring input length
SPAdes
MegaHit
IDBA
ABySS

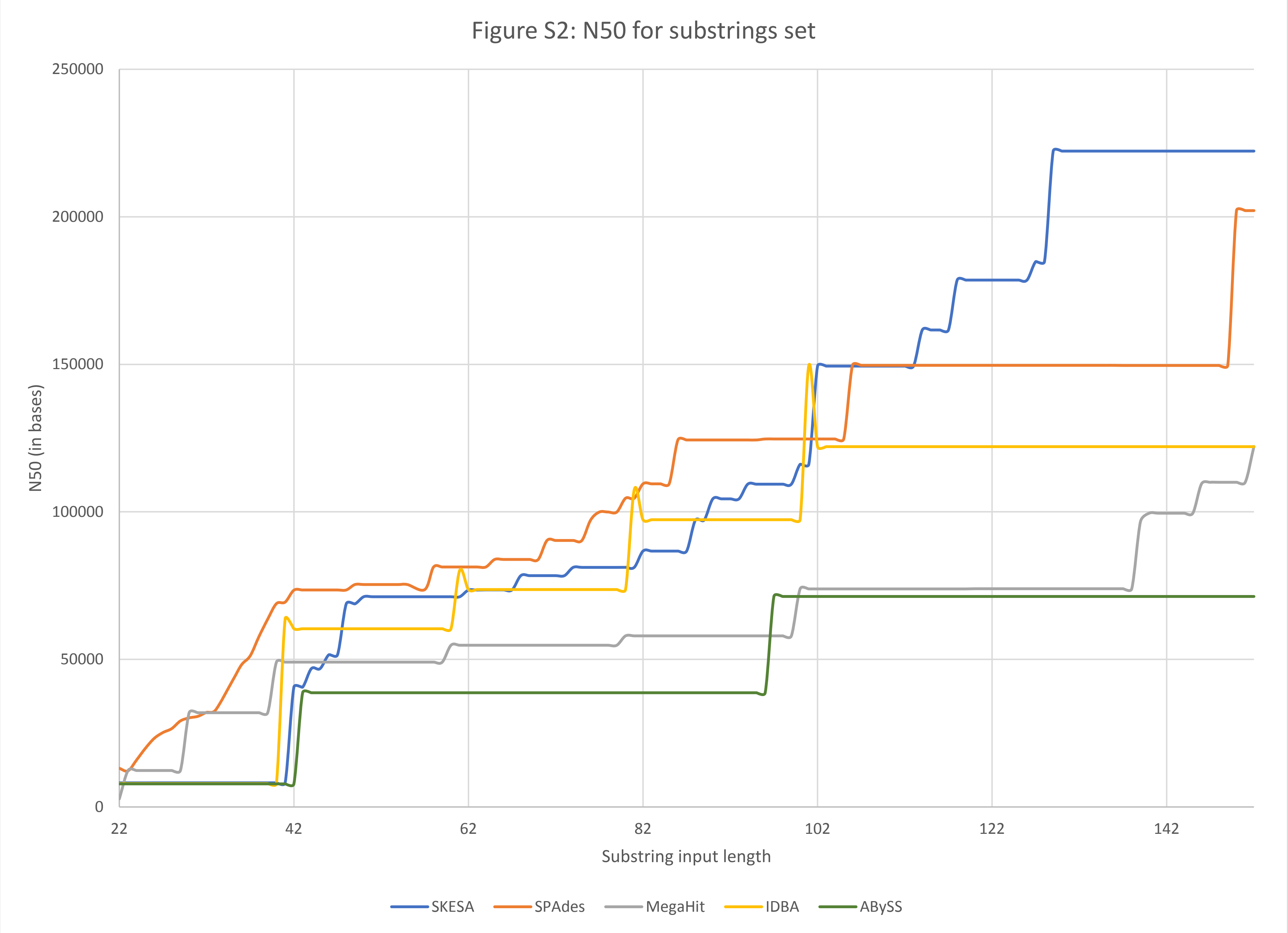

Figure S2: N50 for substrings set
N50 (in bases)
250000
200000
150000
100000
50000
0
22
42
62
82
102
122
142
Substring input length
SKESA
SPAdes
MegaHit
IDBA
ABySS

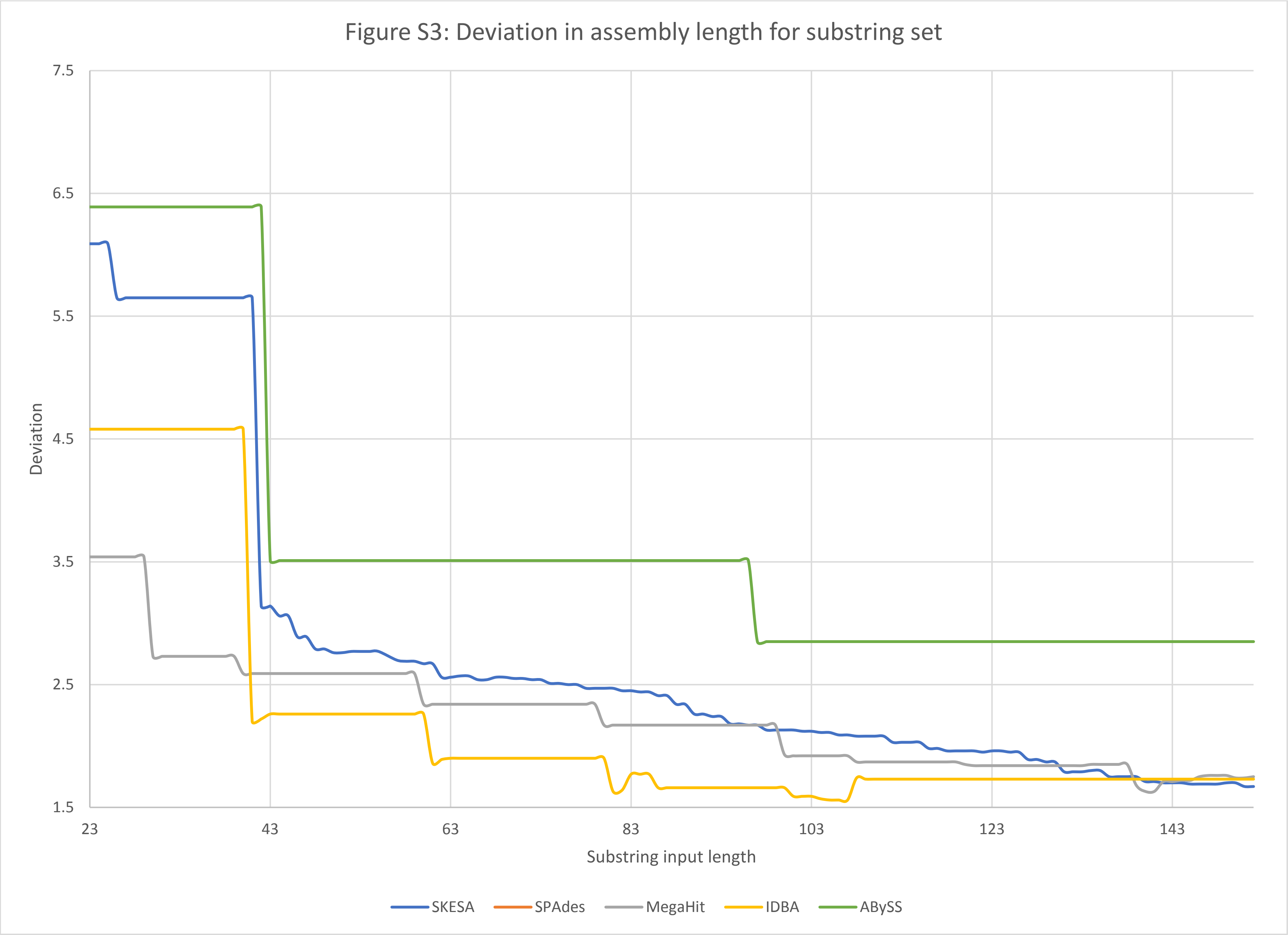
Figure S3: Deviation in assembly length for substring set
7.5
6.5
5.5
4.5
3.5
2.5
1.5
Deviation
23
43
63
83
103
123
143
Substring input length
SKESA
SPAdes
MegaHit
IDBA
ABySS
